# Trust: an essential component in nursing crisis leadership; a hybrid concept analysis

**DOI:** 10.1186/s12912-025-02748-z

**Published:** 2025-01-25

**Authors:** Karin Hugelius

**Affiliations:** Faculty of Medicine and Health, Örebro university, Örebro, Sweden

**Keywords:** Disasters, Emergencies, Leadership, Nursing administration research, Nursing care management, Nursing staff, Trust

## Abstract

**Background:**

Given the increasing trend of disasters, terrorist attacks, pandemics and other crises, crisis leadership is crucial for nurses who lead others and for those working in such situations. There is a need to define and explore the concept of trust as a component of crisis leadership in nursing.

This concept analysis aimed to explore the concept of trust in crisis leadership from a nursing perspective.

**Methods:**

A hybrid concept analysis was conducted. The method consisted of three phases: (I) a theoretical phase relying on a structured literature search, including 11 scientific publications; (II) a field-work phase, in which qualitative thematic analysis of interviews with 30 nurses who had been deployed and/or had led others during crises, was conducted and (III) a final analytic phase, where the results from these data collections were merged.

**Results:**

The analysis suggested that the fundamentals of trust included a perceived intention to do good, the capabilities of both the leader and the team and the perceived predictability of the leader’s behaviour. Trust was found to be built on a perceived forward-looking direction, self-trust and the personal attributes of the leader, such as ethical conduct, the ability to predict the development of crises and an intention to take responsibility and be honest. The social attributes of the relationship between the leader and the team included the intention not to leave anyone behind, loyalty and fostering a sense of belonging among team members. The organisational attributes included a clear organisational structure and clarity of mandate.

**Conclusions:**

Trust is an essential component of crisis leadership that depends on a leader’s perceived intention to do good, predictability of the leader’s behaviour and the capabilities of both the leader and the team. The development of trust relies on the personal attributes of the leader, the social relationship between the leader and the team and organisational attributes. Nurses appointed to lead others during a crisis need to understand the fundamentals of trust as part of leadership in highly demanding situations. Thus, it can be argued that being a leader in a crisis situation requires distinct personal and professional attributes and skills compared to those used to meet routine demands.

**Supplementary Information:**

The online version contains supplementary material available at 10.1186/s12912-025-02748-z.

## Background

Natural disasters, terrorist attacks and/or pandemics occur yearly, causing a significant burden on healthcare services as the number of affected populations continues to increase. In 2023, 93.1 million people were affected by disasters and crises worldwide [1]. Nurses are the largest group of health professionals responding to such crises [2], either as part of their routine work or through temporary deployment. Despite the type and cause of these situations, such events are characterised by a high level of uncertainty, lack of resources, time-critical decision-making and stress [3]. ‘Crisis management’ is a term used in many disciplines, which refers to reducing harm to life, property and the environment [4], and a core component of such management is leadership [5, 6]. Crisis leadership has been defined as leading others during a crisis, as well as preparing organisations to handle crises [7]. Crisis leadership is crucial for both nursing managers in their routine work and nurses temporarily appointed as leaders in such situations. In recent years, not least during the Covid-19 pandemic, the need for crisis leadership ability among nurses and nurse managers has become evident [8, 9].

The concept of crisis leadership refers to theoretical frameworks encompassing cognitive processes, strategic management, gender and role theories and psychological leadership theories [5]. However, despite a growing theoretical base of practice, significant gaps remain in the crisis leadership literature [5]. ‘Trust’ has been identified as one of six core attributes of crisis leadership [10, 11]. Several theories exist on the development of trust as a process over time, such as the integrative model of organisational trust [12], which emphasises individual risk assessment of potential losses and the willingness to be vulnerable by relying on other parties’ actions. Another model relevant to temporary teams is the theory of ‘swift trust’ [13], which develops under specific conditions. According to this model, swift trust develops based on several pre-existing assumptions and the belief that someone is professionally competent to fulfil the duty, function or role they have been assigned. Swift trust, therefore, relies more on assumptions than on personal experience with individual performance developed over time [13]. However, despite agreement on the importance of trust as an essential component in crisis leadership, there is still no consensus or agreed-upon theory or model that explains how such trust should be defined or developed [11]. A starting point for such development is to define and describe the concept of trust as a component of nursing crisis leadership. The aim of this study was to explore the concept of trust in crisis leadership and conceptualise it from a nursing perspective.

## Methods

A hybrid concept analysis in accordance with Schwartz-Barcott and Kim’s hybrid model [14, 15] was conducted. The method consisted of three phases: (I) a theoretical phase, (II) a field-work phase and (III) a final analytical phase.

### (I) Theoretical phase

Step 1. *Select concept:* The concept of trust in crisis leadership was selected.

**Fig. 1:**
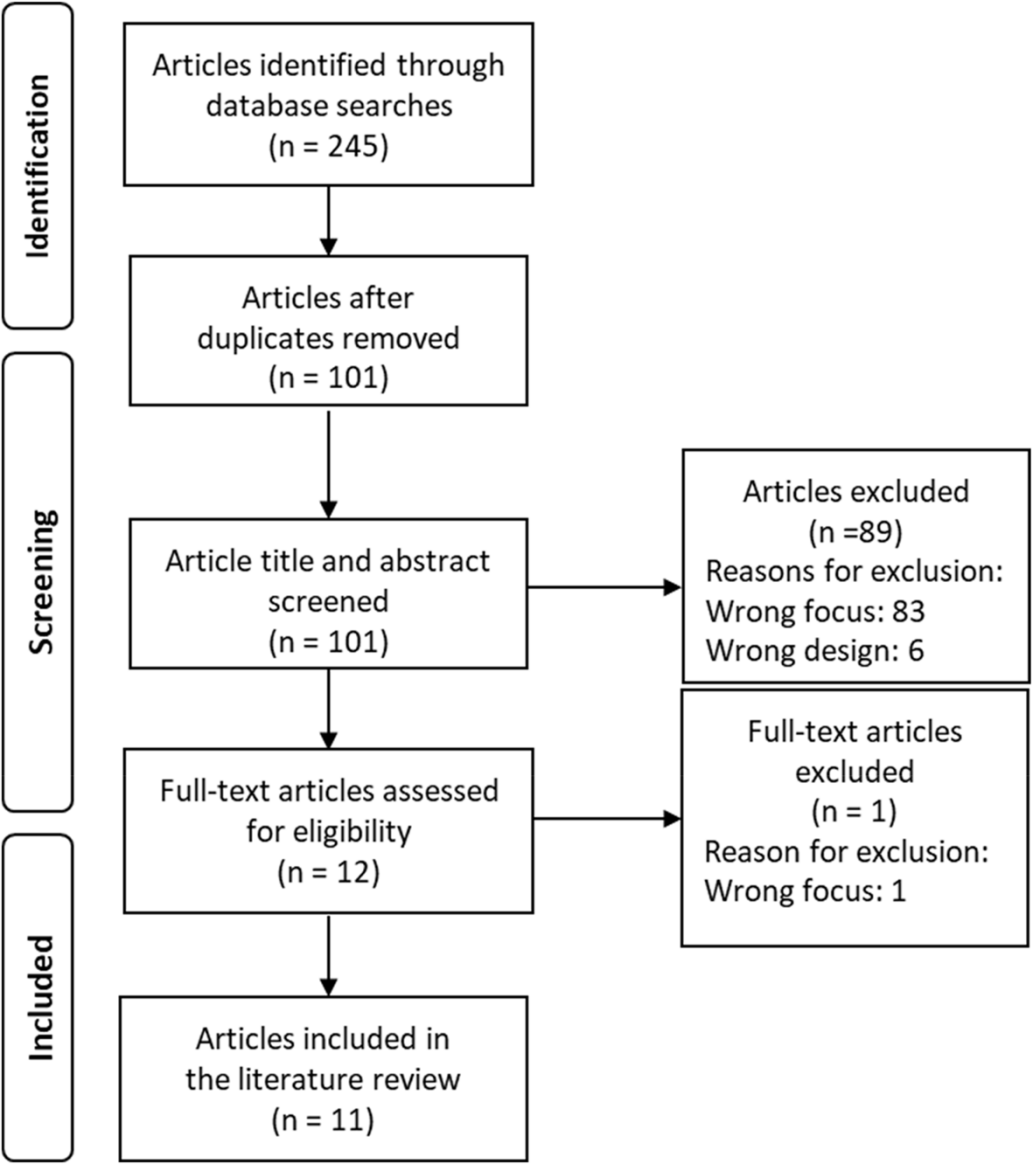
Overview of study selection process

Step 2. *Literature search:* A systematic literature search in PubMed, Web of Science and Scopus was conducted (see Table 1). The search outcome was exported to Covidence for the selection of literature. Papers were included if they related to, described or discussed trust in leadership in any kind of crisis, such as a major incident, disaster or armed conflict and were published in English during the years 2012–2023. Study protocols, editorial texts and reviews were excluded. In all, 101 unique papers were identified. After reviewing the titles and abstracts, 12 were selected for full review. Of these, 11 were deemed acceptable for inclusion in the analysis (see Fig. 1).

**Table 1:** Search strategy

Database and date	Search terms	Number of papers	Title and abstract scanned	Full text scanned	Paper to include
**PubMed** Date for search: September 24, 2023	(((((((disaster[MeSH Terms]) OR (emergencies[MeSH Terms])) OR (mass casualty incident[MeSH Terms])) OR (natural disasters[MeSH Terms])) OR (relief work[MeSH Terms])) OR (rescue work[MeSH Terms])) AND (trust[MeSH Terms]) AND (english[Filter]) Filters: English Sort by: Publication Date	106	106	12	7
**Web of Science** Date for search: December 20, 2023	disaster (Topic) or emergency (Topic) or mass casualty (Topic) AND TS = (leadership) AND TS = (trust) Limits: English, publication year 2012–2023	143	143	5	4
**Scopus** Date for search: January 16, 2024	(TITLE-ABS-KEY disaster (Topic) or emergency (Topic) or mass casualty (Topic) AND TS = (leadership) AND TS = (trust) LIMIT-TO (LANGUAGE, “English”) AND PUBYEAR > 2011 AND PUBYEAR < 2024)	102	102	2	0

Step 3.* Meaning and measurement:* First, the aim, methods and conclusions of each study were mapped (see Table 2). All results related to trust in crisis leadership were extracted and analysed using a thematically inspired approach [16], with the included study results and conclusions sorted by preliminary themes.

**Table 2:** Overview of literature review

Author/s and year	Aim of the study	Study context	Design and methods	Attributes of trust and descriptions of trust in leadership
Brandrud et al. 2017 [17]	To investigate the determinants for the success of EMS as a model for quality improvement in healthcare	Terrorist attack	Qualitative study Focus group interviews with 30 healthcare professionals	Crisis management was based on knowledge, trust and continuous situational awareness analysis. Trust was dependent on continuity, not leaving anyone behind and how rigorously the leader was prepared
Deitchman et al. 2013 [18]	To identify attributes of successful crisis leadership in aviation, public safety, military operations and mining	Public health crises	Literature review and case study	Trust was manifested through maintaining situational awareness, creating team welfare and whether the leader was perceived as experienced. Open communication within the team also contributes to building trust
Hughes et al. 2022 [19]	To explore the impact of Air Force executive nurse leadership on the nursing profession	Military medical leadership	Qualitative method, interview with one person	The leader should show commitment to duty and fairness, and be honest, loyal, have integrity, accept responsibility and cover for the consequences of actions. To gain trust, the leader should praise others in public but reprimand them in private
Hylander et al. 2019 [20]	To describe emergency medical service experiences from real tunnel incidents, as reported by Oslo medical on-scene commanders	Pre-hospital tunnel accidents	Qualitative study, individual interviews with nine pre-hospital medical officers	Trust depended on personal social relations, feeling familiar with the situation and people. By getting to know each other as individuals, a sense of trust was created. Social activities created feelings of trust and built an interest in helping each other
Cao et al. 2022 [21]	To explore the ethical experiences of new nurse managers	The Covid- 19 pandemic	Qualitative study, individual interviews with 19 nursing managers	To be trusted, the managers had to ‘earn’ trust by being tested by their staff. The communication skills and physical and psychological endurance of the leader were essential components of gaining trust. Unfairness led to mistrust and a lack of coherence to instructions given
Jouanne et al. 2017 [22]	To examine elements contributing to the effectiveness of firefighting teams	Rescue operations	Observations of 34 firemen	Organisational trust was related to the team process. However, interpersonal trust was not linked with the team process. Emotional interaction between team members may have a positive influence on team effectiveness and trust
Vasset et al. 2022 [23]	To investigate long-term leader experiences with leader–member exchanges over 25 years	Major incidents and disasters	Qualitative study, interviews with eight health professionals	Trustful relationships between the leader and team were built through dialogue and confirmation of the person. Building trust and respect between people required spending time together
McLaren and Loosemore 2019 [24]	To explore swift trust theory to investigate the process of trust formation within a multinational disaster project management team formed in response to tropical cyclone Winston in Fiji in 2016	Major incident and disaster	Qualitative study, semi-structured interviews with 18 disaster responders and analysis from text documents	Traditional swift trust models need to be contextualised to disaster contexts to add value for such situations. The selection of project team members, based on reputation and formal qualifications, are both critical to the formation of trust.
Phillips et al. 2022 [25]	To explore the experience of leadership and governance during the Covid-19 pandemic from frontline clinicians and stakeholders across the Pacific region	The Covid- 19 pandemic	Mixed methods covering online survey, individual interviews and focus groups	Trust was related to open dialogue, correctness and advocating for oneself and colleagues/partners. Prioritising safety for both employees and the affected population increased the sense of trust
Rosing et al. 2022 [26]	To analyse how action and transition phases produce different task demands for leadership behaviour to enhance trust in the leader and different leader characteristics	Hypothetical leadership styles	Cohort study including 125 firefighters	Different leadership styles were needed to create trust in different phases. Dominant rather than democratic leadership promoted trust in the action phase. In contrast, democratic rather than dominant leadership created trust during transition phases
Tallach & Brohi 2022 [27]	To discuss crisis management and uncertainty	Nursing	Discussion paper	Trust does not occur spontaneously or by luck. Trust is forged through a well-established precedent of roles and leadership

### (II) Field-work phase

Secondary analysis of previously collected data [28] and a new round of study-specific data collection were used to explore the concept of trust in crisis leadership from an empirical nursing perspective.

### First round of empirical data collection

Secondary analysis of previously collected data was based on 27 two-session individual interviews with 15 ambulance nurses [16]. A convenient sample was used, and study participants were recruited through advertisements on social media and emails to ambulance managers in regions in Sweden where major incidents had occurred in the last five years, referring to a webpage where full study information was provided and the opportunity to express interest in participating in the interviews was given. Inclusion criteria were that the nurse should have acted as a pre-hospital incident commander at least one major incident during the last five years. Exclusion criteria were if the person was currently diagnosed with post-traumatic stress or a similar condition related to the specific major incident. The second interview was conducted within two weeks of the first interview, ranging from 7 to 14 days and aimed to follow-up on reflections made by the participants after the first interview. Three participants declined a second interview due to personal circumstances, such as a planned vacation, bringing the total to 27 interviews. The interviews lasted between 2–89 min (first interviews 24–89 min, median 52 min; second interviews 2–22 min, median 5 min). All the nurses had experience being deployed as medical incident commanders at major incidents, such as train accidents, terrorist attacks or large fires [16]. The interviews were conducted by the author (KH) by phone or digital meeting, audio recorded and transcribed verbatim. Secondary analyses of parts relevant to the aim of this study were used.

### Second round of empirical data collection

In the second round of study-specific data collection, three unstructured focus group discussions were conducted with 15 nurses. These nurses had experience as temporarily appointed leaders within a crisis organisation or team, or as daily first-line nursing managers deployed in emergency departments, intensive care units, pre-hospital emergency care units or administrative units The focus group discussions were conducted as part of a disaster medicine course, with voluntary participation indicated by the participants. The participants were arranged into groups of five persons and asked to discuss the question, ‘What do you expect from a leader in a crisis such as a disaster? ‘, with the follow-up questions, ‘What makes you trust the leader? ‘ and ‘How can you build trust as a leader?’ (see Appendix 1). The outcomes of the discussions were shared orally and summarised in writing by the participants. In addition, the facilitator took notes during the discussions. Both oral and written summaries as well as discussion notes were used as data in the analysis.

### Analysis

Data from the two study samples were analysed thematically [15]. The data (transcripts, written summaries and notes from the discussions) were sorted, with guidance from the findings of the literature review analysis, into themes that were thereafter analysed in relation to each other. To verify the analysis from the field-work phase, four study participants from the second study sample were invited and agreed to discuss and verify the findings with the author. These discussions were conducted in an informal way outside the study participants' workplace. First, the author presented the preliminary findings of both the theoretical phase and the results from the field-work data collections. Thereafter, these were discussed with a focus on verifying the interpretations and discussing potential alternative ways of interpreting the results. All participants agreed that the results were valid. The results and analysis process were also discussed during a third-level academic course on concept analysis. Both the research method and preliminary findings from the literature review and field data collections were discussed during seminars. Minor changes in the presentation of the method and results were made as a result of these discussions.

### (III) Final analytic phase

The final step merged the preliminary results from the theoretical phase and the field-work phase in relation to theoretical frameworks.

### Ethical considerations

The data gained from the empirical data collection were reviewed and approved by the Swedish Ethical Review Authority (ref id 2018:232). All participants in the empirical data collections received full written and oral study information, and their consent to participate was registered.

## Results

### Theoretical phase (I)

The semantic description of trust was ‘to believe that someone is good and honest and will not harm you, or that something is safe and reliable’ [29]. Other terms linked with trust in crisis leadership were self-trust, confidence, responsibility, social relations, honesty and competence [17]. An important component of trust is that the leader should be able to be ‘one step ahead’ based on accurate situational awareness [17, 18]. Also, personal relationships and social interaction affected the occurrence of trust between leader and team [20, 23]. Communication skills, attributed to clear and concise information sharing, correctness and an open dialogue, were important to enhance trust [18, 23, 25]. Contextual circumstances, such as the selection of team members [24], spending time together [23], influenced the occurrence of trust in the leadership. However, trust in crisis leadership did not occur by coincidence, but by conscious actions and strategies from the leader [17, 19, 24].

### Field-work phase (II)

In all, 30 nurses (12 males, 13 females) participated in the empirical part of the study. Of these, 10 had experience as first-line nursing managers during crises such as the Covid-19 pandemic, a terrorist attack, a bomb threat against a hospital and a fire at a hospital. Five had experience as nurses deployed in the emergency department or intensive care during the Covid-19 pandemic, a terrorist attack or a fire at a hospital. The remaining 15 had experience as incident commanders and ambulance nurses in major incidents, including train or bus accidents, terrorist attacks and major fires. The participants were aged between 29 and 66 years (median age: 47), with professional experience ranging from 1 to 41 years (median: 26 years). A comprehensive description of each participant is provided in Supplementary file 1.

Five themes were identified: (1) self-trust, (2) personal attributes of the leader, (3) social attributes of the leader-team relationship and (4) organisational attributes and, (5) when trust fails. Together, these elements formed the foundation of trust in crisis leadership, contextualised from a nursing management perspective.

### Self- trust

When deployed as a leader in a disaster, the nurse had to trust in their ability to handle the situation, be confident in contributing to the situation and make the right decisions for others.

‘In such situation there´s no time to hesitate. You must trust yourself, because there is actually no one else to trust….’

To enhance self-trust, the nurses relied on their fundamental leadership competence. It was also expressed that disasters required extraordinary personal and professional skills, along with significant personal demands. The nurses were aware of their appearance, vulnerabilities and personal strengths.

### Personal attributes of the leader

If the leader was perceived by others and themselves as being in control, possessing situational awareness and able to estimate future developments, trust was manifested. Being in control and possessing adequate situational awareness were seen as forming the basis for the leader to make correct decisions. All these abilities were related to the personal competence of the leader. However, competence and skills alone were not enough to build trust. Trust was also dependent on the leader’s intention to ‘do good’ and demonstrate specific behaviours. A leader who appeared balanced, calm, analytical and communicated clearly was deemed trustworthy.

‘She was calm and expressed herself very clearly. That made me trust her, trust that she was on top of this… I could just follow.’

The leadership style required to build trust varied as the crisis developed. Especially in the intensive action phases of a crisis, the leader needed to use more distinct and dominant leadership styles to create trust. The leader should also express the intention to cover for employees’ mistakes or failures, being there for the team members, acting for their well-being and being honest about his/her intentions.

‘Then I trusted myself because I knew that I’m quite good at this. I could rest in the experience and conviction that I was in control.’

The personal attributes also included self-trust, meaning the leader felt capable of managing the situation, grounded in both professional and personal experience, as well as formal training.

### Attributes of the relationship between leader and team members

An important component of trust was the personal relationship between the leader and the team. The context in which the leader and team operated was characterised by unpredictability, insecurity and, most often, an unfamiliar situation for those involved. The team members and the leader were often unfamiliar with each other, but even when they were, the crisis context revealed new sides, traits and competencies not seen in everyday life. Social relations included both professional collaborations and personal relationships, in which team members had to get to know the leader beyond their name and title.

‘We experience a lot in this job. It kind of brings us together in other ways than in a traditional job. Those who you work with, those who are your leader colleagues and your team colleagues, they know a lot about you; what you eat for breakfast, how you smell after some days without a shower and they have seen you at your best and at your worst. They will immediately recognise if you are not honest… and at the same time – it makes me more of a person, not only their boss.’

In addition to deeper personal knowledge, being fair and treating everyone with respect and dignity were deemed crucial to building trust between the team and leader. Continuity, not leaving anyone behind, staying loyal to the team and covering for the team were other attributes deemed necessary. If emotional and personal sharing **–** such as personal circumstances, worries or emotions **–** occurred not only within the team but also between the leader and the team, it increased trust among the members and towards the leader.

‘But if the leader breaks down completely, if he or she loses it, I don’t know, but I wouldn’t trust that person to be able to do the analyses we need and so on. But of course, if he or she shares their worries, shows feelings and even frustration, I think I trust them even more because they are human and I can rely on their inner compass.’

Although it was deemed important for the leader to acknowledge team members and their personal sharing, it was found that a leader should only share their feelings or frustration to a certain degree, while still clearly taking responsibility for the team, its leadership and themselves, in order not to lose the members’ trust. If the leader acknowledged both individual and team efforts, it further reinforced the trust relationship between the team and leader.

### Organisational attributes

A clear organisational structure, in which the parts had defined roles and mandates, enabled trust, while unclear mandates or roles undermined trust.

‘It was such a mess… I didn’t understand why we didn’t have a clear organisation with mandates and clear communication lines. That made it so difficult to trust him.’

Other aspects of organisational attributes that affected trust included gaining official recognition for the efforts made during crises. This recognition should be provided at the strategic or political management levels. Moreover, a clear forward-looking perspective on the overall operation was seen as important. The organisation should not get stuck on past details or mistakes but should show respect for the complexity of the situation.

‘You need a balance of control and formalised mandates. However, so many unpredictable things will likely happen, and in those moments, you need to act or make decisions and rely on those above you to trust you. Otherwise, I cannot continue trusting the team either.’

The organisational structure and mandates needed to allow a certain degree of freedom to act independently, though still under the purview of higher management. Since the situational conditions were constantly changing, not all decisions or actions could be sanctioned beforehand. Therefore, a clear mandate and trust from the highest management level were essential for leadership at the operational level.

### When trust fails

If the situation and leadership were characterised by a high level of uncertainty regarding the consequences of the event, mistrust manifested. Furthermore, if the leader was perceived as incompetent to lead or withheld facts from the employees, mistrust manifested.

‘If you say one thing and do another… you can go home. No one will trust you anymore.’

A lack of transparency regarding facts, as well as the leader not acting in accordance with their declared intentions, resulted in mistrust. The same occurred if the organisation at the strategic management level did not cover the actions and decisions of operational leaders. This created insecurity and heightened the level of uncertainty, leading to stress and mistrust within the team and between the team and leader.

### Final analytic phase (III): Towards a description of trust in crisis leadership

**Fig. 2:**
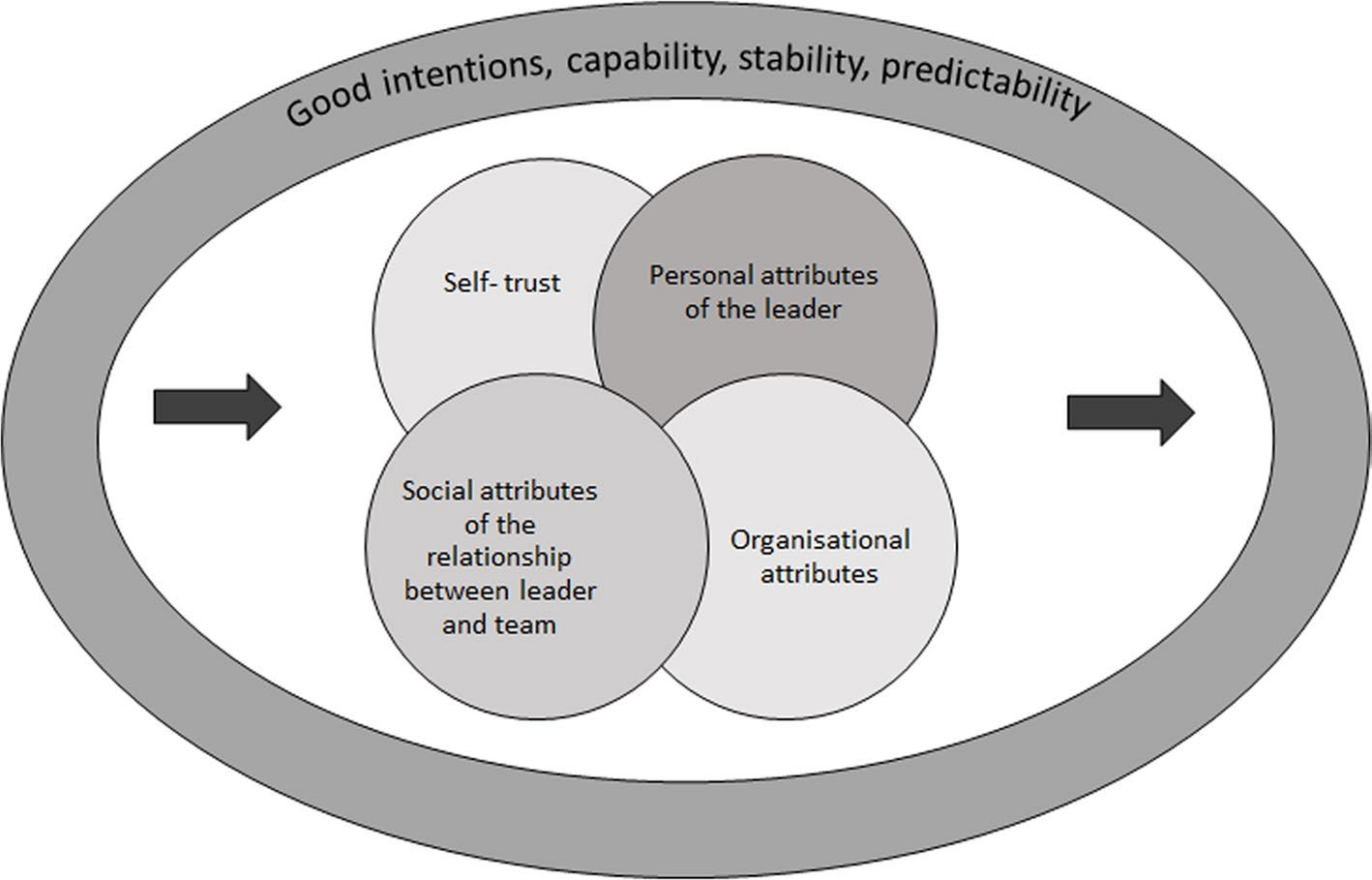
Illustration of trust in crises leadership

Upon merging the preliminary results from both the theoretical and field phases, a comprehensive understanding of trust consisting of four components were identified: (1) self-trust, (2) personal attributes of the leader, (3) social attributes of the leader-team relationship and (4) organisational attributes. The identified fundamentals of trust were a perceived intention to do good, competence, stability, recognition and predictability. Trust, as a component of crisis leadership, was dependent on self-trust, the personal attributes of the leader **–** such as personal behaviour, the ability to predict developments, and the intention to take responsibility. The social attributes of the relationship between the leader and the team that engendered trust encompassed the intention to include everyone, not leave anyone behind, transparency of information and create a sense of belonging and loyalty among team members. The organisational attributes that engendered trust included a clear organisational structure and clarity of roles and mandates as well as a clear intention to cover the actions and decisions of the leader. These aspects contributed to the manifestation of trust and were mutually dependent; none could be excluded (see Fig. 2). Moreover, trust was built on an estimated and perceived forward direction rather than a retrospective perspective relying on the previous empirical experience of the leader or the team members.

## Discussion

This hybrid concept analysis has shown that trust in crisis leadership in nursing depends on the self-trust of the leader, the personal attributes of the nurse as a leader, the social relationship between the leader and the team and organisational attributes. Trust is also dependent on the perceived intention of the leader to do good, the capabilities of both the leader and the team, and the perceived predictability of the leader’s behaviour.

Previous research on crisis leadership has primarily focused on the skills and behaviours required to manage the relationships necessary to lead others [30]. However, the findings of this study indicate that the personal skills of the leader do not fully explain how trust between the team and the leader, or between different management levels, develops or can be sustained during a crisis. The personal attributes and skills required of the leader must be considered within a context in which the team has certain responsibilities, with organisational perspectives **–** including mandates and roles **–** being fundamental. Previous studies have highlighted the lack of research addressing the causal relationship between contextual factors, such as the task, team and individuals on the team [30]. In a crisis, political, structural and cultural contextual factors also need to be considered to align leadership competence with each crisis event [30].

One factor influencing crisis leadership is time [31]. A crisis will always develop dynamically and, in most cases, will affect healthcare services for longer and in different ways than initially expected [3, 32]. Therefore, different leadership styles are needed to create trust in different phases of the crisis. A dominant leadership style may help build trust during the response phase, but in the transition or recovery phase, democratic leadership contributes to trust [26]. In a crisis, decisions must be based on good planning, luck, training and previously established trust [27]. Interpersonal trust is most often based on previous personal knowledge or judgements of others [33]. This suggests that trust requires time. However, in a crisis, the response team often consists of individuals who are not familiar with one another, and the deployment may be temporary and brief. Based on both the literature review and empirical data, this study revealed that trust can also be based on immediate impressions or preconceptions. These impressions may stem from rumours, personal reputation or a perceived forward-looking direction. This finding suggests that the immediate impression of the leader by team members plays a crucial role in developing trust within an operational crisis management organisation. This finding also raises the question of how trust between the team and its leader is affected by long-term crises or an unsuccessful response. In everyday nursing leadership, the attributes of trust have been described at both organisational and personal levels [34]. However, the social relationships between the leader and team, identified in this study as essential for enabling trust, have not been previously described. Likewise, there are similarities between the attributes of trust in general nursing leadership and crisis leadership, such as the importance of perceived predictability and open communication.

A term used to refer to enhanced trust in ordinary nursing leadership is ‘trustworthiness’ [34, 35]. However, none of the study participants mentioned trustworthiness when describing trust in the context of a crisis. One possible explanation is that the crisis context involves high uncertainty and dynamic development, making it difficult to assess the quality of messages or information, as crises inherently involve constantly changing circumstances. Consequently, it becomes difficult to judge trustworthiness. Another explanation is that operational crisis management situations are usually short-term compared to ordinary nursing leadership, and trustworthiness plays a more significant role in trust development over time than in acute settings where transparency and information sharing might play a more important role to build trust.

Authentic leadership is a style associated with increased trust in the leader [36]. Authentic leadership relies on four key components: self-awareness, relationship transparency, balanced decision-making and moral standards. Moreover, it depends significantly on self-awareness and self-development in both the leader and team members [36]. This leadership concept can lead to better working environments, more positive healthcare staff and optimal patient outcomes [37]. Authentic leadership, however, does not specifically pertain to crisis situations. In crises, a nurse must cross environmental, professional and personal borders and is frequently exposed to extreme human suffering and potentially dangerous situations [38, 39]. Another difference in crisis response, compared to everyday nursing management, is that disaster or crisis response often involves working in temporary team constellations [39] and under constantly changing and unpredictable circumstances. However, the intentions of authentic leadership **–** to help nurses find meaning and optimism, promote resilience and foster healthy working environments [36] **–** are also highly desirable in crises, where physical and psychosocial demands may be overwhelming [39]. In such situations, the need for clear structures and mandates may outweigh the importance of the leadership style used in everyday management. Therefore, this study supports the idea that crisis leadership differs from everyday leadership within nursing, even though the foundation remains the same. It can also be argued that being a leader in a crisis requires somewhat different personal attributes and skills compared to ordinary management duties.

The reviewed literature relies on studies of trust during crises, viewed from the perspective of the healthcare system or medical crises. However, this was not part of the selection criteria, and some studies also reported findings from other sectors, such as rescue services. One question that needs addressing is whether the character or manifestation of trust in a crisis response team differs based on the cultural context of the services **–** such as nursing or healthcare services **–** or whether trust in crisis leadership shares the same attributes and meaning across all sectors. Are the attributes of trust within an emergency department, the police or a military unit different from or similar to the processes of gaining trust in crisis leadership? Most of the papers identified in the structured literature search were published recently. This suggests an increased interest in trust as an aspect of crisis leadership. However, there is still an urgent need for deeper understanding of how trust is manifested and lost, as well as how training and mental preparedness for disaster nursing managers can be optimised. This study focused on trust as a component of operational crisis leadership. However, the relationship between trust in operational leadership (e.g., that exercised by a first-line nursing manager or team leader), trust at strategic or political management levels and how these processes interact with first-line leadership requires further research.

## Limitations

The hybrid concept analysis method combines the rigour of empirical observation with theoretical analysis, merging the strengths of both approaches [40]. In this paper, the literature search likely did not identify all available papers on the topic, as only 11 relevant papers were found. The literature search was not limited to papers on trust in crisis leadership within nursing but included all forms of operational crisis leadership. On one hand, this can be seen as an advantage, as the phenomenon of trust likely does not differ by context, allowing the inclusion of more papers. On the other hand, it can be seen as a limitation, as all social or psychological concepts must be conceptualised to add value to practice. Another limitation of the concept analysis methodology is the lack of a formal quality assessment of the included papers [40, 41].

Other concept analysis methods recommend including at least 30 papers to achieve a sufficiently substantial number [41]. On the other hand, if few papers are published on a topic, the hybrid concept analysis method may be suitable, as it combines theoretical and empirical sources to verify results. In this study, the empirical data consisted of two separate data collections in different nursing contexts, providing broad insight into the phenomena that complemented the literature review. The literature search includes papers from a global perspective, while the field phase includes only Swedish nurses and nursing managers. The national cultural aspects of how trust in leadership may be viewed are important and could be a next step for further investigation. To account for potential cultural or geographical biases, combining both a systematic literature search and empirical data may add value. No contradictions between the literature review results and the empirical findings were observed, adding validity to the results.

A major limitation of this concept analysis is that it was conducted by a single author. However, a rigorous methodology can reduce the biases inherent in studies by a single author. Additionally, this study was initiated during a course in concept analysis, and critical friend reviews from six individuals were used to improve the quality of the analysis and results. The critical friend concept was used to stimulate constructive critical dialogue to promote both personal and professional development and self-reflection. The process was similar to a review process but also included aspects of personal and professional reflection [42]. However, these critical friends did not meet the Vancouver criteria for authorship and were therefore not declared as authors.

## Conclusion

Trust is a crucial component of crisis leadership, dependent on a perceived leader's intention to do good, the predictability of the leader’s behaviour and the capabilities of both the leader and the team. The development of trust depends on the leader’s personal attributes, the social relationship between the leader and the team and organisational attributes.

The ability to quickly establish trust between the leader and the team is a fundamental component of crisis leadership. Nurses appointed to lead others during a crisis need to understand the fundamentals of trust as part of leadership in highly demanding situations characterised by high levels of uncertainty and significant human suffering. Trust in crisis leadership differs in some respects from everyday nursing leadership; thus, being a leader in a crisis requires somewhat different personal and professional attributes and skills compared to those used in daily leadership demands. As scientific knowledge regarding trust as a component of crisis leadership remains limited, further empirical studies exploring this topic are recommended.

## Supplementary Information

Supplementary Material 1.

## Data Availability

The data used to support the findings of this study are available upon request from the author.
