# Supplementary material for: Trust: an essential component in nursing crisis leadership; a hybrid concept analysis
Source: BMC Nurs. 2025 Jan 25;24:91. doi: 10.1186/s12912-025-02748-z (PMC11762076; doi:10.1186/s12912-025-02748-z)
Supplement: Supplementary file 1 — Supplementary Material 1. [file 12912_2025_2748_MOESM1_ESM.docx]

| Supplementary file 1. Overview of study participants demography | | | | | |
| --- | --- | --- | --- | --- | --- |
| **Study parti-cipant** | **Gender** | **Age in years** | **Professional experience in years** | **Role within the crises management**  **system** | **Type of crises event experience** |
| 1 | Female | 41 | 13 | First-line nursing manager | Hospital fire |
| 2 | Female | 36 | 22 | First-line nursing manager | Pandemic |
| 3 | Female | 48 | 21 | First-line nursing manager | Terrorist attack |
| 4 | Female | 56 | 26 | First-line nursing manager | Pandemic |
| 5 | Female | 45 | 12 | First-line nursing manager | Bomb threat to hospital |
| 6 | Male | 62 | 39 | First-line nursing manager | Terrorist attack |
| 7 | Male | 45 | 12 | First-line nursing manager | Terrorist attack |
| 8 | Male | 59 | 13 | First-line nursing manager | Pandemic |
| 9 | Male | 49 | 26 | First-line nursing manager | Pandemic |
| 10 | Male | 55 | 30 | First-line nursing manager | Pandemic |
| 11 | Female | 34 | 14 | Clinical nurse | Pandemic |
| 12 | Female | 29 | 15 | Clinical nurse | Pandemic |
| 13 | Female | 41 | 20 | Clinical nurse | Hospital fire |
| 14 | Male | 49 | 23 | Clinical nurse | Pandemic |
| 15 | Male | 51 | 28 | Clinical nurse | Terrorist attack |
| 16 | Female | 32 | 18 | Prehospital incident commander | Train- bus collision |
| 17 | Female | 29 | 1 | Prehospital incident commander | Terrorist truck attack |
| 18 | Female | 52 | 17 | Prehospital incident commander | Bus accident |
| 19 | Female | 29 | 3 | Prehospital incident commander | Terrorist truck attack |
| 20 | Female | 41 | 19 | Prehospital incident commander | Traffic accident |
| 21 | Male | 37 | 16 | Prehospital incident commander | Bridge collapse |
| 22 | Male | 62 | 39 | Prehospital incident commander | Terrorist truck attack |
| 23 | Male | 38 | 22 | Prehospital incident commander | Fire at an elderly home |
| 24 | Male | 51 | 29 | Prehospital incident commander | Bus accident |
| 25 | Male | 38 | 18 | Prehospital incident commander | Bus accident |
| 26 | Male | 51 | 26 | Prehospital incident commander | Bridge collapse |
| 27 | Male | 36 | 12 | Prehospital incident commander | Explosion |
| 28 | Male | 41 | 6 | Prehospital incident commander | Bus accident |
| 29 | Male | 61 | 29 | Prehospital incident commander | Traffick accident |
| 30 | Male | 43 | 11 | Prehospital incident commander | Chemical incident |
